# Enhanced Acoustic Adhesion Enabling Agile and Small Wall‐Climbing Robot Design With Anisotropic Origami Structure

**DOI:** 10.1002/advs.78084

**Published:** 2026-10-14

**Authors:** Xinxian Chen, Kanglong Yuan, Ao Qin, Yikun Liu, Yusen Ma, Jun Peng

**Affiliations:** ^1^ School of Mechanical Engineering Tsinghua University Beijing China; ^2^ School of Mechanical Engineering Xi'an Jiaotong University Xi'an China

**Keywords:** acoustic adhesion, inspection robot, origami spring, visual identification, wall‐climbing robot

## Abstract

Small inspection robots are highly desirable for detecting damage within confined spaces. By vibrating a disk on a surface, a thin air layer with negative pressure—known as acoustic adhesion—is generated, which offers a strategy for creating compact yet strongly climbing robots. However, it is highly sensitive to disturbances. Any transmitted vibrations or improper torque can compromise the robot's airtightness, causing it to fall. This fatal flaw has hindered the development of reliable acoustic climbing robots. Here, we present a 28 g, 50 mm inspection robot capable of navigating vertical walls (8 cm/s) and ceilings (20 cm/s) while carrying a 100 g payload. We decouple vibration and load effects using a ring connection, approximately doubling the adhesion. Moreover, an anisotropic origami spring is designed to maintain close contact between the disk and the substrate, ensuring durable, robust adhesion. By combining acoustic adhesion with optimized structural designs, we overcome two major challenges: failure from vibration coupling and demanding assembly. Our robot thereby achieves high mobility and a large payload capacity. We demonstrate the robot's agility and intelligence through maze navigation and inspection of real blades, in which it autonomously adjusts its path. This work offers a potential solution for in‐situ damage detection.

## Graphical abstract

This 28 g robot incorporates innovative ring structures and anisotropic origami springs to achieve high speeds and exceptional load capacity. It successfully carries a 100 g payload while ascending walls or ceilings at speeds of up to 8 and 20 cm/s, demonstrating enhanced adhesion and maneuverability over current climbing robots.

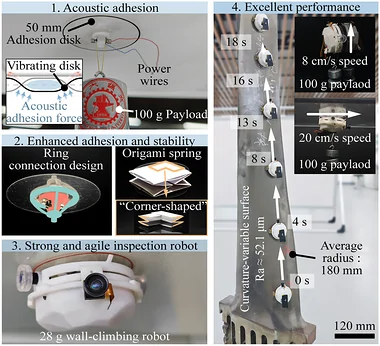

## 1 Introduction

Climbing inspection robots, capable of navigating vertical and inverted surfaces, have expanded the range of inspection tasks and demonstrated considerable potential across fields such as industrial equipment monitoring and disaster relief operations. Adhesion capability is a core design determinant, as it directly dictates the driving mechanisms, overall dimensions, and load‐bearing capacity of the system.

A variety of adhesion methods have been explored in the literature, including dry adhesion [1, 2, 3, 4], electrostatic adhesion [5, 6, 7, 8], magnetic adhesion [9, 10, 11], and negative pressure adhesion [12, 13]. Biomimetic dry adhesion, for instance, draws inspiration from the microstructure of gecko setae [14, 15, 16, 17], where van der Waals interactions enable robust attachment to vertical or inverted surfaces. Although this approach can sustain considerable loads, it is susceptible to surface contamination. Electrostatic and magnetic adhesion techniques—relying on electro‐adhesion disks or permanent magnets and solenoids [6, 18, 19], respectively—can produce strong adhesive forces. Still, they require substantial energy: electrostatic systems often operate at voltages exceeding 5 kV, while magnetic systems may require over 5 W to power solenoids. Meanwhile, robots inspired by suckerfish or octopuses use negative‐pressure adhesion, powered by air pumps or external devices that evacuate air and create a vacuum seal [20]. Despite offering impressive adhesion, such systems are often bulky, which limits their practical deployment.

To overcome the limitations of conventional negative‐pressure methods, recent research has focused on more compact and energy‐efficient alternatives. Wang et al. [21] integrated a vibration motor into a vacuum cup to generate centrifugal‐force‐induced suction. Similarly, a vibrating disk has been shown to form adhesion under external excitation [22, 23], a phenomenon explained by gas lubrication theory, in which a thin air layer forms between the disk and the substrate, producing a negative‐pressure field [22, 23, 24, 25]. This type of anisotropic adhesion—characterized by high normal strength and low tangential resistance—is particularly suitable for climbing robot applications. Weston‐Dawkes and colleagues developed an 8‐cm‐diameter acoustic adhesion robot capable of traversing walls at 3 cm/s. However, this early work was exploratory and demonstrated only basic locomotion without a defined application context. In a complementary approach, Jia's team leveraged the tangential centrifugal force generated by a vibrating motor to develop an ultra‐thin robotic disk. This minimalist design enables secure adhesion and locomotion across inverted surfaces with minimal components. K. Yuan et al. designed and fabricated a two‐segment acoustical adhesion wall‐climbing robot that can traverse wall corners via its mechanical structure [26].

Wall‐climbing robots based on acoustic adhesion achieve higher locomotion speeds and superior load‐to‐weight ratios than conventional wall‐climbing robots. The key to this advantage lies in the formation of a negative‐pressure air layer between the acoustic adhesion module and the substrate, which generates an air‐cushion lubrication effect akin to that of air‐cushion vehicles. The resulting lubrication markedly reduces tangential resistance, with experimental results showing a ratio of normal adhesion force to tangential resistance as high as 300:1. As a result, such robots combine high mobility with a high load‐to‐weight ratio that is competitive with pump‐driven negative‐pressure systems.

However, generating a stable negative‐pressure air layer requires stringent geometric conditions: the vibrating disk must remain parallel to the substrate, with a micrometer‐scale gap consistently maintained. Consequently, when a single vibrating disk is integrated on a robot, assembly inaccuracies and surface irregularities can severely degrade adhesion performance. Meanwhile, vibrations can be transmitted to the robot body through improper connection structures, inducing coupling effects that destabilize the negative‐pressure air layer and reduce the maximum adhesive force achievable with this method. These two issues have severely limited the performance and practicality of acoustic adhesion. This work introduces an origami‐inspired spring and a specialized ring connection to address these challenges, yielding an intelligent climbing robot with high payload capacity and rapid mobility. The origami springs leverage mechanical anisotropy to deliver robust, consistent adhesive force even under external disturbances. Simultaneously, the ring connection optimizes stress distribution across the disk, decouples vibration from load transfer, and prevents air leakage. Equipped with this enhanced adhesion disk, the robot demonstrates exceptional load‐bearing capability and agile locomotion on both walls and ceilings. Furthermore, it incorporates an onboard camera for environmental inspection and a microcontroller that communicates with a host computer running a custom recognition network, enabling targeted inspection and adaptive motion control. These integrated functionalities render the wirelessly controlled microrobot highly suitable for applications such as equipment cavity inspection and in situ non‐destructive testing.

## 2 Results

### 2.1 Design Principles of Wall‐Climbing Inspection Microrobots

Strong and stable adhesion is critical to the design of wall‐climbing robots. Driven by a vibration motor, a vibrating disk periodically draws in and expels air at the disk–substrate interface. A thin air layer with negative pressure forms as more air is continuously squeezed out (Figure 1A). Inspired by the phenomenon of near‐field acoustic levitation, ultrasonic vibration can carry a 1.7 kg plate without contact [27, 28, 29, 30]. We refer to this adhesion as acoustic adhesion, which requires both mechanical vibrations and a micrometer‐scale air layer.

**FIGURE 1:**
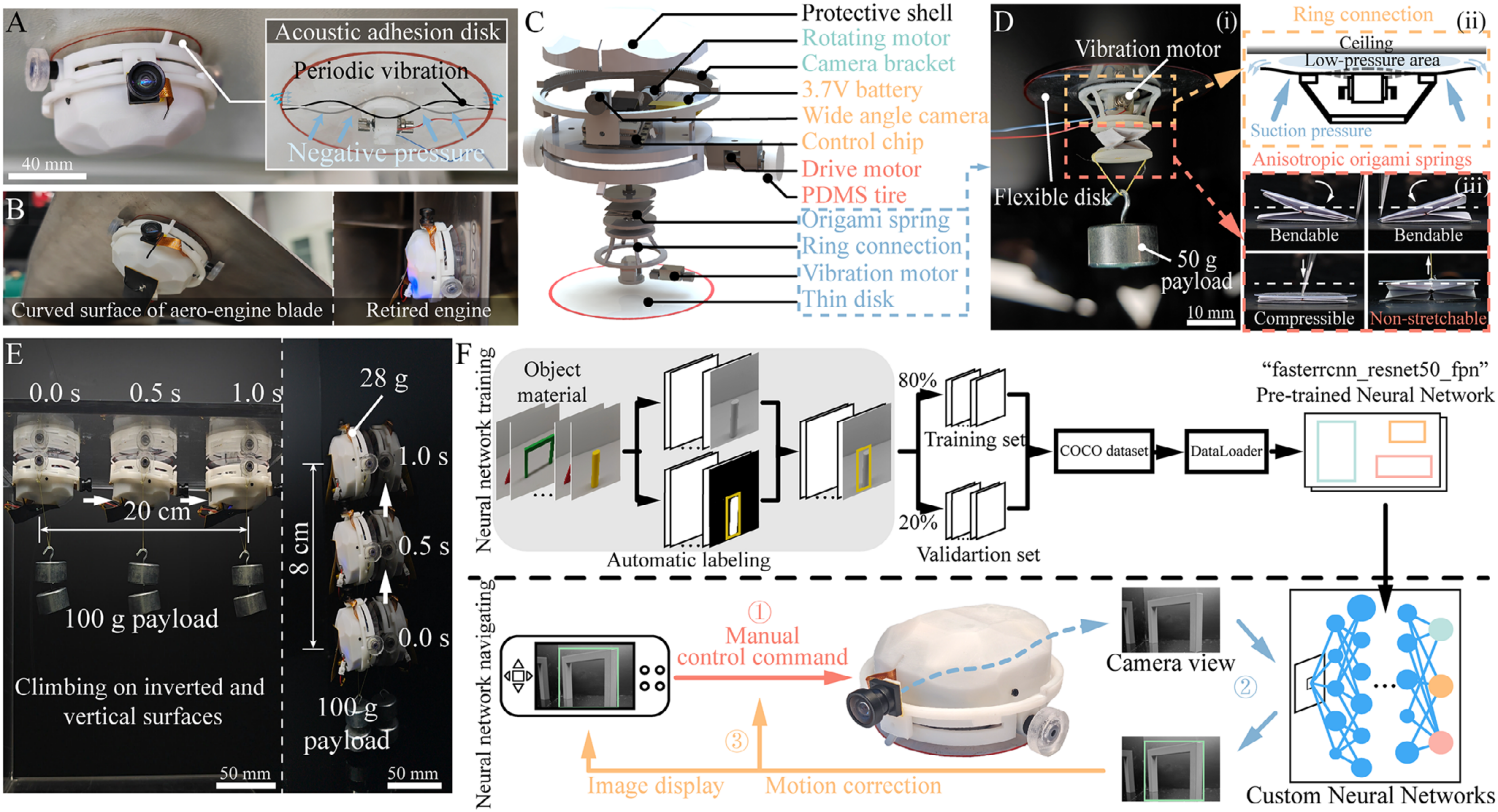
An intelligent wall‐climbing microrobot. (A) The robot climbs on the ceiling of an aluminum plate using an acoustic adhesion disk. The disk is periodically vibrated by an external excitation (such as a vibrating motor), continuously squeezing air out and creating negative pressure. (B) The robot climbs on curved surfaces of real engines thanks to a flexible adhesion disk. (C) The diagram of the robot structure. (D) The anisotropic adhesion system comprises a ring connection and an origami spring. The ring‐connection design balances gravity‐induced loading and adhesive vibration to enhance adhesion performance. The anisotropic origami springs ensure the vibration disk obtains a uniform air layer over the substrate, leading to stable adhesion. (E) This 28 g robot climbs on inverted and vertical polymer surfaces with a 100 g payload at 20 cm/s and 8 cm/s, respectively. (F) A schematic illustration of the robot's intelligence system. The robot utilizes the hue‐saturation‐value (HSV) color space for large‐scale automatic labeling of recognized objects. These labeled images are then fed into the pre‐trained neural network (*Faster‐RCNN*) for further training, resulting in a customized object recognition model. This neural network enables microrobots to analyze captured photos, identify objects, and adjust moving paths. Microrobots could use image recognition to detect damage in confined machine cavities rapidly.

Although acoustic adhesion demonstrates excellent adhesion performance, it has not been widely adopted because it requires stable vibration, and any disturbance can cause detachment. Thus, a properly designed adhesion disk is expected to decouple vibration and adhesion, and an anisotropic connection spring is required to compensate for the robot's assembly errors and to fit curved surfaces.

We designed and assembled a 28 g, 50 mm climbing microrobot, as shown in Figure 1A. The adhesion module enables stable attachment to the substrate, while the drive wheels provide bidirectional locomotion and perform zero‐radius turns on walls and ceilings. To control this robot and detect its environment, an intelligent control unit (embedded within the shell) comprising an antenna, a microchip, and a camera unit with a rotating motor is responsible for responding to human motion commands, processing visual data, and coordinating the robot's movements accordingly. The strong adhesion and intelligence enable microrobots to agilely climb on various surfaces, including the curved surfaces of aero‐engine blades and retired engine blades (Figure 1B). The flexible adhesion disk can conform to curved surfaces. Exploded drawings of detailed structures are shown in Figure 1C. The green components are the camera rotation unit (see Text S1 for more details); the orange components are the controller feedback unit; the red components are the drive unit; and the blue components are the adhesion unit. Detailed component weights are shown in Table S1.

Adhesion is fundamental to wall‐climbing performance. We propose an enhanced acoustic‐adhesion module in Figure 1D(i). To overcome the vibration‐load coupling problem in acoustic adhesion, the ring connection pulls the disk at different radii, as shown in Figure 1D(ii). At the same time, the negative pressure acts as an adhesive force, preventing the disk from detaching from the substrate. In this case, the vibration and load forces are absorbed by different components, thus avoiding interference between them. The desired external load is applied along the disk's axis to verify that the force is uniform and to prevent any bending that could compromise the airtightness. However, in practice, due to substrate unevenness or assembly errors, the load force and the disk are not perfectly perpendicular. This inspired the use of an origami mechanism, leading to the design of an anisotropic origami spring to address this issue, as shown in Figure 1D(iii). The spring can withstand tensile forces without transmitting bending, thereby ensuring that the force acting on the disk does not weaken its adhesive effect.

The robot achieved strong climbing performance, enabled by enhanced acoustic adhesion and refined structural design. It moved with a 100 g payload at 20 cm/s on the ceiling and 8 cm/s on the wall, respectively, in Figure 1E and Movie S1. This speed is equivalent to 3 times the self‐length per second. For comparison, the gecko is 1.8 times its self‐length per second. The high load capacity further accommodates onboard electronics, including the microcontroller and battery required for wireless control and video transmission.

A custom neural network was developed to identify obstacles and correct moving paths (Figure 1F). First, a video of the object to be recognized was recorded, and frames were extracted using OpenCV. To avoid the time‐ and labor‐intensive process of manual contour annotation, we used the hue‐saturation‐value (HSV) color model to automatically label contours, reducing processing time from 17 min to 8 s. We then retained the bounding box labels and desaturated the image to encourage the neural network to focus on shape rather than color, as the object's color in real‐world environments may not always be distinct. This labeling method offers a framework for universal visual recognition, eliminating the need for extensive manual annotation and addressing the limitations of color‐based object recognition.

These labels were superimposed on the decolorized image to form the training data, which was then imported into the pre‐trained model Faster R‐CNN [31] to obtain the customized neural network. While the robot is controlled for movement and inspection, the camera captures the surroundings in real time and transmits the video stream to the computer via Wi‐Fi. The customized neural network analyzes the images, labels the objects, and corrects the movement by adding a turn of 20% or less, then sends the data back to the controller for display. For the specific process, refer to Text S2. This intelligent path‐correction system enhances the wall‐climbing robot's autonomous mobility, enabling it to rapidly reach target locations within the custom‐designed maze (Figure 4E). The robot successfully navigates to predetermined waypoints while avoiding obstacles, completing the task in only 13 s, thereby highlighting the potential of vision‐guided control for wall‐climbing robots.

### 2.2 Decoupled Adhesive Vibration from Load

The controllable vibration of the disk is triggered by a motor (operating at nearly 200 Hz), generating a transient negative pressure for robot climbing (Figure 2A). The stable negative pressure results from periodic membrane vibrations, which induce rapid gas exchange between the gap and the external environment, thereby creating a sustained pressure differential, as illustrated in Figure 2B. It is worth noting that the adhesion disk exhibits low tangential friction because an air layer separates it from the substrate. This characteristic makes the acoustic adhesion disk well‐suited for wheel robots.

**FIGURE 2:**
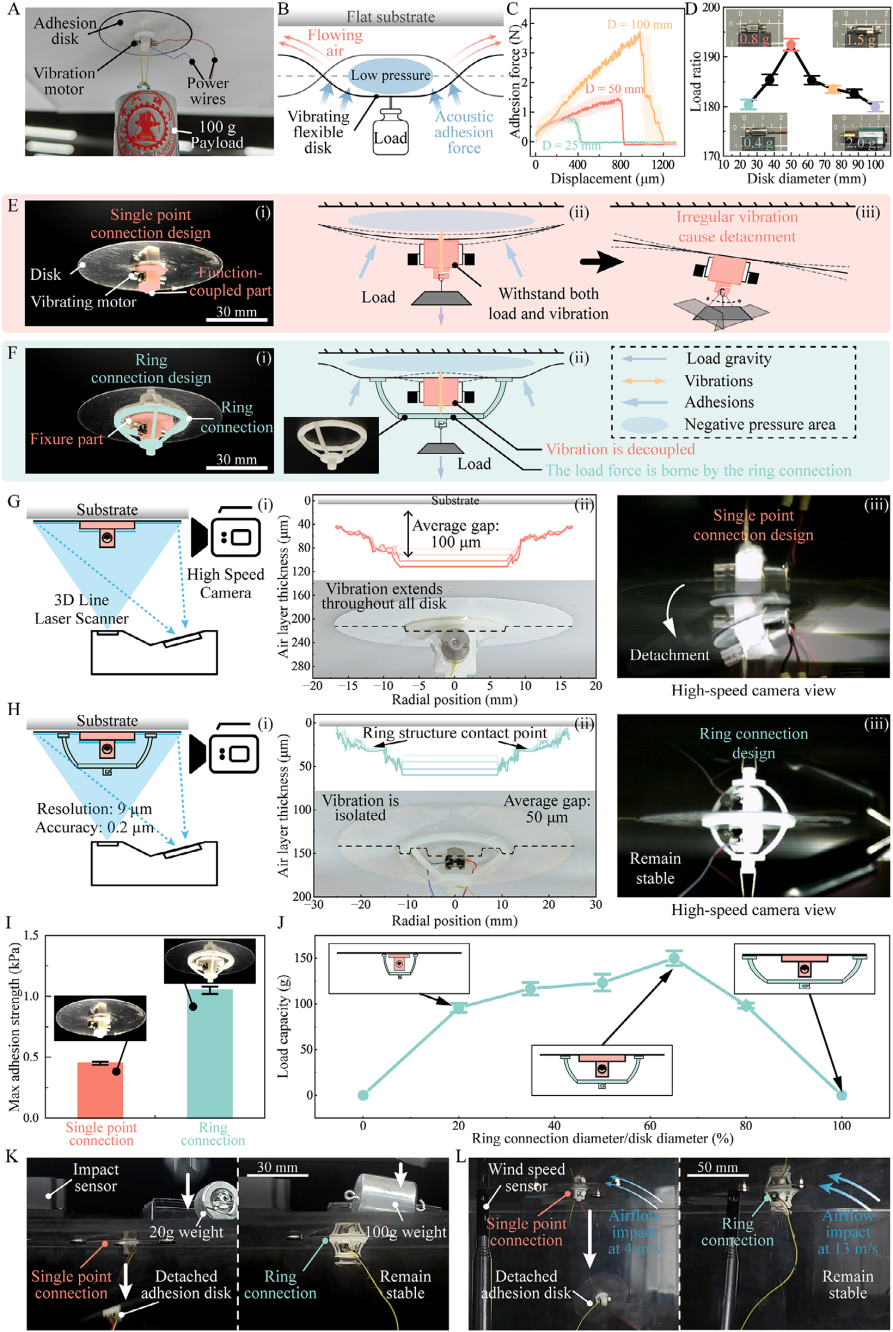
Enhanced adhesion performance with the design of the ring connection. (A) Schematic illustration of the acoustic adhesion mechanism. Driven by a vibration motor, the disk vibrates, inducing periodic air intake and expulsion. As more air is progressively expelled, a negative‐pressure air layer forms beneath the vibrating disk. This results in anisotropic adhesion characterized by a strong normal vacuum force and minimal tangential friction, which is sufficiently low to be considered negligible. (B) Schematic representation of acoustic adhesion generated through dynamic vibrations. Under appropriate disk dimensions and vibration frequencies, the disk undergoes periodic compression, creating a cavity with negative pressure relative to the ambient environment. (C) Normal adhesion force was evaluated via tensile testing, in which disks of varying diameters were pulled away from the substrate. Results indicate that larger disk diameters are associated with higher adhesion forces. (D) Load‐ratio performance of adhesive devices with different disk diameters and matched vibration motors. As larger disks require more powerful vibration sources for excitation—increasing energy consumption without proportional gains in adhesive force—the load ratio decreases. Thus, the 50 mm disk was chosen to optimize loading efficiency. (E) The single‐point‐connection design disk and force diagram. (i) An image of a single‐point‐connection disk. (ii) The starting state of the single‐point‐connection disk. The disk is subjected to vibration, creating a negative pressure zone that generates adhesion to resist the gravitational load. (iii) Detachment occurs due to unstable loads. (F) Ring‐connection disk and force diagram. (i) An image of a ring‐connection disk. (ii) Decoupling principle of the ring‐connection disk. A ring connection is used to carry the load force, ensuring it is not affected by vibration and remains stable under a greater payload. (G) Schematic of the single‐point‐connection design for air gap thickness evaluation. (i) Setup for measuring the thin air layer beneath the disk using a 3D line laser scanner and a high‐speed camera. (ii) Dynamic distribution of air layer thickness during half a vibration cycle. (iii) High‐speed camera sequence showing disk adhesion and detachment in the single‐point configuration. (H) Schematic of the ring‐connection design for air gap thickness assessment. (i) Measurement configuration for the thin air layer under the disk via a 3D line laser scanner and high‐speed camera. (ii) Variation in air layer thickness over half a vibration cycle. (iii) High‐speed footage of the ring‐connection adhesion disk. The optimized connection structure enables sustained adhesion. (I) The stable maximum load for the two disks. The ring‐connection disk carries a greater weight, specifically twice that of the single‐point‐connection disk. (J) Influence of ring connection diameter on adhesive force. (K) Comparison of single‐point‐connected and ring‐connected adhesion disks under impact disturbances. The ring‐connected configuration decouples vibrations from the payload, exhibits a smaller air gap, withstands more severe impact disturbances, and demonstrates greater robustness. (L) Compared with single‐point‐connected adhesion disks, ring‐connected adhesion disks exhibit greater resistance to airflow‐induced disturbances and maintain stable adhesion at flow velocities of up to 13 m/s.

We examined the variation in adhesive force during the peeling of disks of three different diameters from their substrates using a tensile testing machine; detailed information—such as substrate material, surface roughness, and vibration frequency, among other parameters—is provided in the Methods section. The results demonstrate that the adhesive force increases with greater stretching, thereby providing more space for vertical disk vibration and generating a more substantial vacuum effect. Moreover, larger disk diameters yielded higher maximum adhesive forces, as illustrated in Figure 2C. This observation is consistent with previous studies. However, the use of larger disks demands higher excitation energy, necessitating the incorporation of larger vibration motors. This, in turn, reduces the adhesion device's load ratio. In effect, a greater portion of the adhesive force is expended on supporting the device's own weight rather than the external load, thereby compromising overall efficiency. We evaluated the load ratios across multiple adhesion devices, each combining a specific disk diameter with a corresponding vibration motor. The results demonstrate that the adhesion device attains its maximum load ratio—and thus optimal efficiency—when equipped with a 50 mm disk diameter, as indicated by the red point in Figure 2D.

Therefore, a 50‐millimeter‐diameter disk was selected as the basis for subsequent optimization. The vibration motor mentioned earlier is connected to the disk through a custom‐designed part. In prior designs, such connections typically employ a single‐point‐connection structure (Figure 2E(i)), which uses a multifunctional component to transmit both vibration and load, resulting in coupling that affects adhesive performance, as illustrated in Figure 2E(ii). This multifunctional part holds the vibration motor to the disk and conducts the vibration to it. The robot body is connected to this part, applying load forces to it. However, the disk is continuously deformed by vibration and load, preventing it from returning to its original position. This causes irregular load oscillations and an increased air gap thickness, eventually leading to a loss of airtightness and abrupt detachment, as shown in Figure 2E(iii).

To mitigate this coupling, we designed a ring connection to decouple disk excitation from external loading, as shown in Figure 2F(i). The vibration motor is fixed to the disk by the fixture component; however, this part no longer transmits the load force from the robot body. Instead, the ring connection takes over this function, as depicted in Figure 2F(ii). This structure effectively decouples the forces acting on the adhesion disks and improves adhesion performance by regulating vibration amplitude, thereby eliminating potential interference between vibrational and load forces. Furthermore, the ring connection distributes the applied load over a larger area, lowering local contact stresses and avoiding the concentrated loading inherent to single‐point interfaces.

We quantified the air‐gap thickness for adhesion disks with single‐point and ring connections using a 3D line‐laser scanner and a high‐speed camera (Figure 2G(i) and H(i)). The results demonstrate that, in the single‐point‐connection design, variations in air gap thickness (Figure 2G(ii)) indicate that the structure is simultaneously subjected to tensile and vibrational loads, causing vibrations to propagate across the entire disk. Concurrently, the presence of relatively large internal voids (∼100 µm) induces irregular oscillations in the load‐bearing components. Furthermore, the edge regions are susceptible to inadequate sealing, increasing the risk of detachment. High‐speed camera recordings further corroborate that the vibrations are transmitted through the connection components to the entire adhesion disk, resulting in pronounced, unstable amplitude fluctuations that ultimately lead to failure, as shown in Figure 2G(iii). In contrast, the ring connection design confines vibrations to the central region because it absorbs all tensile forces. This configuration decouples tensile and vibrational forces, allowing them to act independently, and ultimately reduces the air layer thickness to approximately 50 µm—about half that of the single‐point design in Figure 2H(ii). This finding aligns with the finite element simulation results shown in Figure S1. This improvement enhances the negative‐pressure sealing effect of the adhesion disk, enabling sustained, stable adhesion, as shown in Figure 2H(iii) and Movie S2.

We compared the single‐point‐connection disk and the ring‐connection disk to quantify their maximum stable adhesion performance, as shown in Figure 2I and Movie S2. This demonstrates that the ring‐connection adhesion disk exhibits more stable vibrations and supports approximately twice the weight of the single‐point‐connection disk.

The diameter of the ring connection plays a critical role in determining both the range of vibration suppression and the effectiveness of the flexible sealing at the edges. To quantify this dependence, we measured adhesion force as a function of diameter for different ring connection structures, as shown in Figure 2J. A ring structure with a minimum diameter leads to stress concentration on the disk, preventing the full utilization of the remaining surface area. On the other hand, an overly large diameter compromises or even negates the adaptive, flexible sealing effect at the periphery, thereby impeding the maintenance of the internal negative‐pressure chamber and ultimately causing detachment. Optimal adhesion performance is achieved when the ring structure diameter is approximately 70% of the adhesion disk diameter. We propose that this specific configuration strikes a balance between mitigating stress concentration and preserving the flexibility required for effective edge sealing. Moreover, during disk vibration, these particular points along the disk's diameter coincide with a vibration node—a phenomenon consistent with prior research [24, 32]. Anchoring the ring structure at this location minimizes interference with vibrational behavior.

Moreover, the ring connection enhances the adhesion disk's resistance to disturbances. To quantify this improvement, impact and airflow disturbance tests were conducted to simulate casing vibration and internal exhaust disturbances commonly encountered during engine blade inspections.

The results showed that the single‐point‐connection adhesion disk fell when subjected to an impact from a 20 g object, as shown in Figure 2K and Movie S3; the measured impact acceleration was 0.48 m/s^2^, and the impact displacement was 53 µm within half a cycle of acoustic‐adhesion vibration. Considering that the edge air layer thickness of the single‐point‐connection disk at rest was approximately 40 µm, the cumulative edge air layer thickness under impact reached approximately 100 µm. This prevented a seal from holding, leading to rapid air loss from the internal negative‐pressure region and consequent detachment. In contrast, the ring‐connection adhesion disk remained stable under a 100 g impact, with an impact acceleration of 1.97 m/s^2^ and an impact displacement of 83 µm over half a cycle of acoustic‐adhesion vibration. Owing to the vibration–load decoupling inherent in the ring connection, the edge air layer thickness was only approximately 2 µm, offering a substantial margin. As a result, the disk withstood greater impacts while remaining stable; its cumulative edge air‐layer thickness during impacts was approximately 85 µm, sufficient to maintain the seal.

Under airflow disturbances, the single‐point‐connection adhesion disk detached when subjected to a 4 m/s airflow, primarily due to the Bernoulli effect and airflow intrusion into the negative‐pressure zone, as shown in Figure 2L. In contrast, the ring‐connection adhesion disk, featuring a tighter edge seal, resisted greater airflow intrusion and the Bernoulli effect, remaining stable even under an airflow of 13 m/s.

These two experiments demonstrate that the ring connection enhances the robustness of acoustic adhesion, providing the adhesion disk with greater fault tolerance and enabling it to remain stable under disturbances.

### 2.3 Origami‐Inspired Anisotropic Spring

The acoustic adhesion disk exhibits exceptional adhesive performance. However, the adhesion disk has limited tolerance for excessive torque. When the disk is subjected to torque beyond its design capacity, bending deformation occurs at one end of the disk, compromising the airtight seal and ultimately leading to detachment, as illustrated in the upper section of Figure 3A. Such over‐torque situations are frequently encountered in robotic applications because assembly tolerances can shift the center of mass of the body or payload. In light of this, the connection between the adhesion disk and the robot body must possess specific attributes. It requires high tensile and shear strength to effectively transmit adhesive force, while exhibiting low compressive and bending stiffness so that assembly tolerances and body‐induced torques do not degrade sealing or adhesion. These intricate constraints limit the suitability of a range of isotropic, flexible connection materials and structures—such as sponge and silicone—for this project.

**FIGURE 3:**
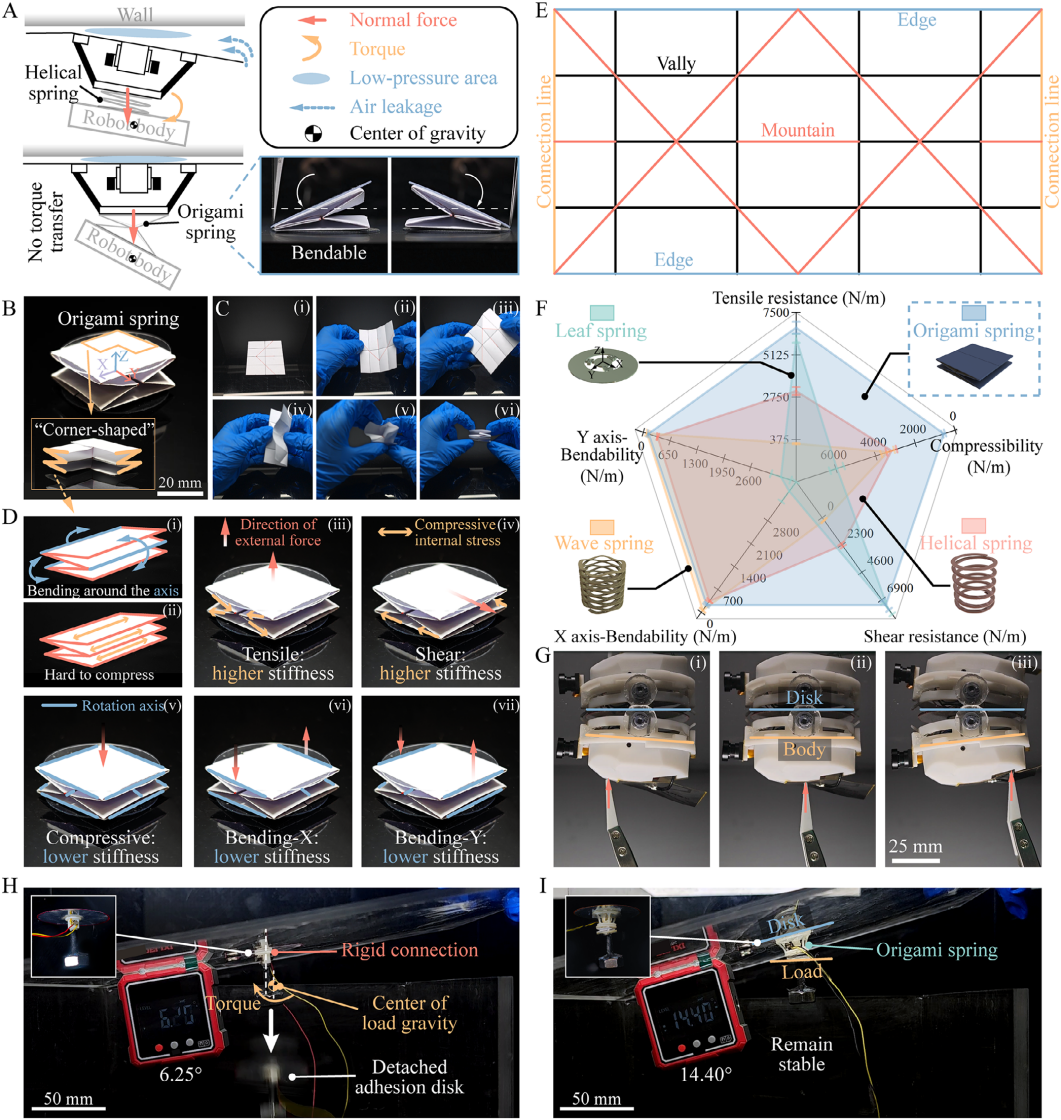
Improved adhesion using origami‐inspired anisotropic springs. (A) Diagram of the effect of different springs on the adhesion disk. The conventional helical spring transfers torque from the body to the adhesion disk, and the tilted disk causes adhesion failure. In contrast, deformable origami springs can absorb the bending torque, ensuring stable adhesion. (B) Images of an origami spring, with an inset of a quarter section. (C) The folding process of a quarter section of the origami spring. (D) Schematic diagrams of various forcing directions. (E) The complete crease diagram of the origami spring. (F) Comparisons of balanced mechanical properties of different kinds of springs, normalized to each other. The origami springs exhibit the most balanced performance under diverse mechanical conditions. (G) The microrobot equipped with an origami spring exhibited stable adhesion and remained attached during the tested tweezer perturbations. (H) Experimental diagram illustrating the detachment of a rigidly connected adhesion disk at a small inclination angle. (I) Inclination experiment employing an origami‐spring adhesion disk. Owing to the anisotropy of the origami spring, angular deviations can be compensated, thereby preventing the application of torque to the acoustic adhesion disk. Consequently, adhesion is maintained even at an inclination angle of 14°.

To address this challenge, we designed an origami‐inspired anisotropic spring, as depicted in Figure 3B. This origami spring is bendable, thereby preventing torque transfer to the adhesion disk, as shown in the lower part of Figure 3A. It is formed by folding four “corner‐shaped” structures (see the inset of Figure 3B) from a single sheet of paper and connecting the head and tail, as described in Figure 3C and Movie S4.

Inspired by simple multi‐layered folded structures commonly found in daily life, such as corrugated cardboard and the veins of canna lily leaves, we designed an “E‐shape” origami unit, as illustrated in Figure 3D(i) and Figure S2. This “E‐shaped” origami unit exhibits structural anisotropy. It incorporates three blue creases that permit rotation about all three axes, enabling substantial compression as shown in Figure 3D(i). At the same time, the acute‐angle configuration formed by these creases effectively resists pressure along the X‐axis, as depicted in Figure 3D(ii). Moreover, to prevent misalignment between the robot and the adhesion disk during motion, we specifically aimed to restrict tangential deformation along the Y‐axis. To achieve this, we folded two “E‐shaped” structures vertically to form a single “corner‐shaped” unit. Notably, this design also provides high tensile stiffness while maintaining low compressive stiffness. This is because, during tensile loading, the structure tends to unfold into a parallel sheet, creating strong resistance at the perpendicular intersections of the “E‐shaped” sides, as shown in Figure 3D(iii). Since the adhesion disk is circular, an axisymmetric spring layout was desired. Accordingly, we arranged the four “corner‐shaped” structures in a rotationally symmetric pattern.

As a result, the new origami spring demonstrates anisotropic mechanical properties, with high tensile stiffness (Figure 3D(iii)) and high XY shear stiffness (Figure 3D(iv)), while retaining low stiffness in other directions (Figure 3D(v, vi)). This enables the spring—whose full crease pattern is shown in Figure 3 E—to conform effectively to substrate inclinations, ensuring a snug and airtight fit. These characteristics match the mechanical requirements of the climbing robot.

In contrast, conventional springs, such as helical, wave, and leaf springs, cannot provide the required anisotropic performance due to mismatches in their design purposes. To benchmark the mechanical advantages of the origami spring, we experimentally quantified stiffness along five loading directions and compared it with representative conventional springs (Figure 3F). The values in the figure represent the deformation stiffness in each direction. The results are displayed in Figure 3F using different colors: green for leaf springs, blue for origami springs, orange for wave springs, and red for helical springs. The more uniform the area, the better the overall performance; any depression in a specific area indicates that the spring's performance is unsuitable for wall‐climbing robots.

Both leaf springs (green) and wave springs (orange) exhibit notable incompatibility. The leaf springs' limited bendability and compressibility make it difficult for the robot to adapt to the substrate, and they are susceptible to assembly errors. Meanwhile, wave springs lack sufficient stiffness in tension and shear, weakening their ability to transmit adhesion. In contrast to these two springs, helical springs (red) provide a well‐balanced performance across the tested directions.

However, the origami springs (blue) demonstrate superior deformation properties in compressibility and bendability, enabled by folding along prescribed creases. The origami springs also exhibit higher stiffness in tension and shear, as the stress is not aligned with the creases. Specific tensile‐force data are shown in Figure S3. This provides a more comprehensive and superior solution, making them ideal for use as connection components. Therefore, the microrobot, equipped with origami springs, exhibits stable, strong adhesion even under random poking (Figure 3G and Movie S4).

To quantitatively evaluate the tilt‐compensation capability of the origami spring, two configurations of adhesion disks were tested. Rigidly connected adhesion disks, representing connection mechanisms with high bending stiffness, such as coil springs, wave springs, and leaf springs, transmit the torque induced by displacement of the center of gravity to the adhesion disk under tilted conditions. Consequently, the edge on one side of the disk was pried up, resulting in rapid air leakage and eventual detachment, as illustrated in Figure 3H and Movie S3. In the experiment, the center of gravity was displaced by 5 mm, generating a torque of 0.6 mN m and thereby compromising the sealing performance. By contrast, when positioned on an inclined substrate, the origami spring underwent bending deformation owing to its anisotropic mechanical properties, as shown in Figure 3I. This deformation suppressed torque transmission to the adhesion disk and improved its conformability to the substrate. Because assembly deviations may occur between the adhesion disk and the substrate to which the robot attaches, and the substrate may be inclined, the origami spring reduces the acoustic adhesion disk's sensitivity to angular variations, thereby enhancing the robustness of the adhesion system.

### 2.4 Agile Movement and Intelligent Inspection

Our robot is a two‐wheeled mobile robot shown in Figure 4A, which uses the difference in rotational speed between the two wheels to steer. We assume the rotational speeds of the two wheels are *v*_1_ and *v*_2_ respectively, the direction vector of the robot is expressed as:

(1) $$\left\{\begin{array}{c} \omega =\frac{v_{i}-v_{2}}{H}\\ v=\omega R=\frac{v_{1}+v_{2}}{2}\\ R=\frac{H\left(v_{1}+v_{2}\right)}{2\left(v_{1}-v_{2}\right)} \end{array}\right.$$

where *R*, *H*, *v*, and ω mean turning radius, wheelbase, forward speed, and spinning speed, respectively. We precisely control each wheel's speed, enabling the robot to move agilely. The robot's payload capacity on the wall is mainly dependent on wheel friction in Figure 4A, which is calculated using the following formula:

(2) $$G_{robot}+G_{payload}\leq F_{adhesion}\cdot \mu$$

where μ means the friction factor between the wheel and wall. Acoustic adhesion relies on the continuous intake and expulsion of air, forming a lubricating gas layer between the membrane and the substrate. As a result, friction forces between the adhesion device and the substrate remain on the order of millinewtons—sufficiently low to be considered negligible in this context. The formula indicates that, in addition to adhesion force, the wheel friction factor also affects load capacity. Therefore, we manufactured PDMS soft tires to enhance friction and enable our robot to climb on various surfaces, such as engine blades and frosted glass, as shown in Figure 4B. Notably, on vertical walls, the adhesion force primarily manifests as normal pressure on the wheels, and the motor torque characteristics govern the maximum load. Considering the inverse speed‐torque relationship inherent in compact motors, we selected a 30 gf cm torque motor with a 160‐rpm capacity in this work (see Text S3), based on a systematic analysis of mobility and payload requirements.

**FIGURE 4:**
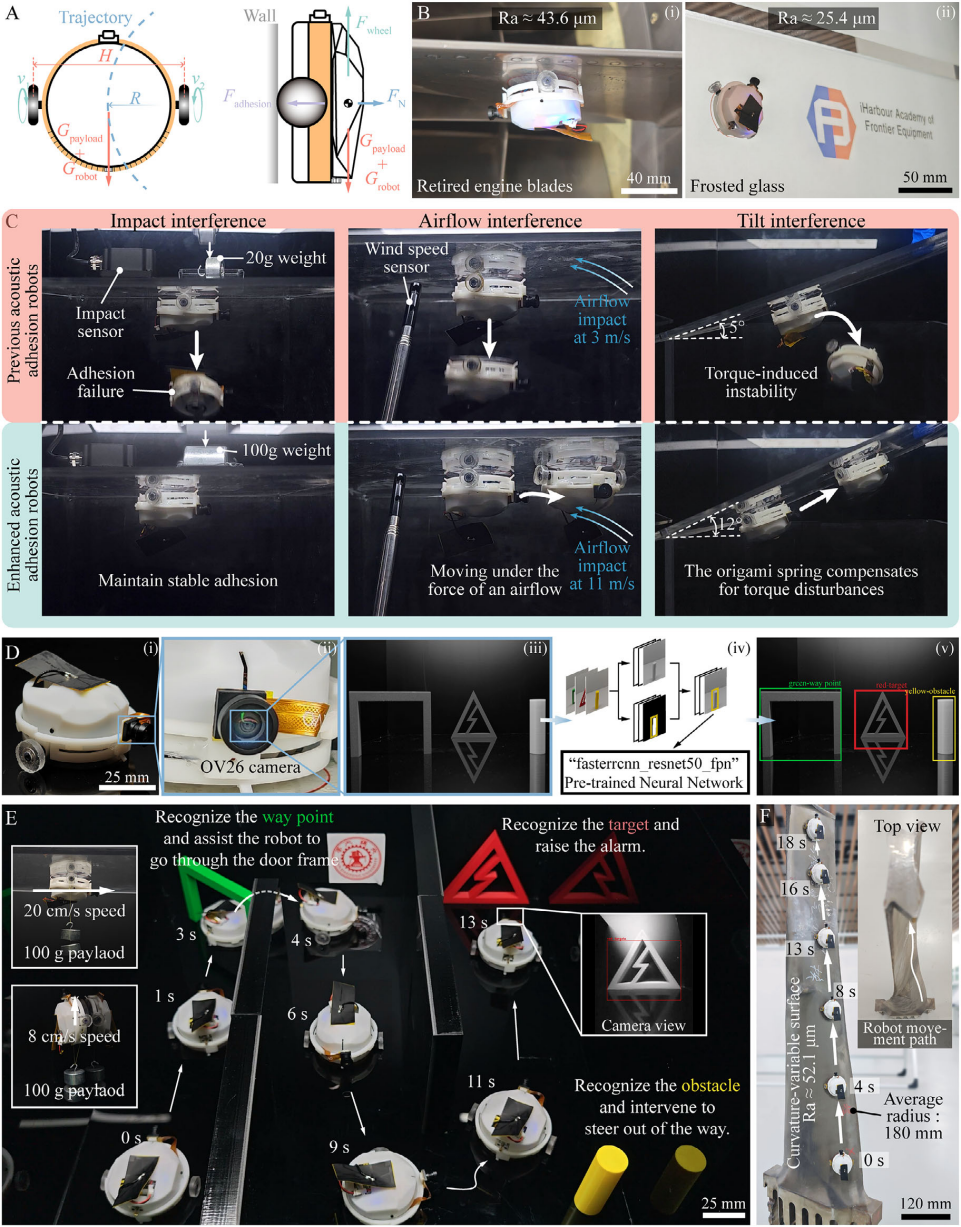
Demonstration of agile movements with real‐time feedback of visual recognition and adaptability to complex surfaces. (A) Schematic of the differential speeds of wheels during complete turns and force analysis when climbing on vertical walls. The robot's weight (including the robot and payload) is balanced by the friction from the wheels, which depends on the normal adhesion force. (B) Images of a robot climbing on turbine blades and grooved, rough glass. (C) A comparison of wall‐climbing robots equipped with the previous acoustic adhesion disk and the enhanced acoustic adhesion disk under various disturbance conditions. The proposed robot (green area at the bottom) exhibits substantially improved robustness against impact, airflow, and tilt disturbances compared with the wall‐climbing robot equipped with the previous acoustic adhesion disk (red area at the top). (D) The workflow of visual recognition and inspections. The camera captures an image, and the advanced processor performs preliminary processing, transferring the image to the computer for further processing. Custom‐trained neural networks, using datasets generated by automated HSV color annotation, enable efficient object recognition. (E) The robot can carry a 100 g payload at 20 cm/s on the ceiling and at 8 cm/s on the wall, as demonstrated in a maze. The intelligent assistance enables the robot to successfully navigate through the green door frame, avoid the yellow square, and locate the red sign in 13 s. (F) The microrobot climbs the complex surfaces of steam turbine blades using a flexible adhesion disk.

The key issues hindering the development of acoustic adhesion—namely, vibration–load coupling and excessive sensitivity to torque—are identified and validated in this paper, and a ring connection and an origami spring are specifically designed to decouple the forces and compensate for tilting. This advancement enhances the load‐carrying capacity of acoustic‐adhesion wall‐climbing robots and improves their robustness, thereby conferring genuine practical potential. To demonstrate the improved disturbance resistance of the enhanced acoustic‐adhesion disk, two wall‐climbing robots were constructed and subjected to disturbances (representative of common disturbance types encountered in internal inspections of engine blades), as shown in Figure 4C and Movie S3. The upper red area in the figure depicts a robot equipped with the previous‐generation acoustic‐adhesion disk (employing a single‐point‐connection structure and a leaf spring). In contrast, the lower green area depicts a robot equipped with the enhanced acoustic‐adhesion disk (employing a ring connection and an origami spring). When subjected to impact, airflow, and tilting disturbances, the robots designed in this work withstood greater disturbances while maintaining stable adhesion and movement, demonstrating that the enhanced acoustic‐adhesion disk has overcome prior limitations and offers improved stability and practical applicability.

The decoupled acoustic adhesion disk and origami spring design provide excellent adaptability for wall climbing, allowing the robot to carry various sensors and chips. To enhance its capabilities, the robot is equipped with a wide‐angle camera and an advanced processor, as shown in Figure 4D. The OV2640 camera captures real‐time images of the surrounding environment. Additionally, the advanced processor chip performs initial image processing, including stabilization, desaturation, and video streaming. The processed video is then transmitted to a computer via Wi‐Fi. Custom‐trained neural networks, utilizing datasets generated through automatic annotation in the HSV color space, enable efficient object recognition and feedback to the robot. This approach, which uses the HSV color space for automatic annotation, simplifies neural network development and enables real‐time, high‐precision motion correction. For the specific process, refer to Text S2.

We constructed a maze to demonstrate the robot's performance, as shown in Figure 4E and Movie S5. The green door frame, yellow square, and red lightning bolt represent waypoints, obstacles, and the target to be detected, respectively. The robot started from the designated start line, captured images, and identified the three objects. It intelligently incorporated a 20% or less turn into its motion module to assist navigation and displayed the corresponding information on the controller. The robot successfully passed through the green door frame, avoided the yellow square, and located the red lightning bolt. This entire operation took just 13 s, showcasing the robot's excellent agility and highlighting its potential for intelligent maintenance and search‐and‐rescue applications. Meanwhile, the robot can carry a 100 g payload at 20 cm/s on the ceiling and 8 cm/s on the wall, as shown in the left insets of Figure 4E. This indicates that the robot can transport repair modules, such as laser torches, during inspections.

Owing to the anisotropic origami spring and the decoupled acoustic adhesion disk, this wall‐climbing robot demonstrates remarkable adaptability to a variety of surfaces. It is capable of maintaining stable adhesion and locomotion across diverse substrate materials, varying degrees of surface roughness—including metal turbine blades and frosted glass depicted in Figure 4 B—as well as complex curvatures, such as those of variable‐curvature turbine blades illustrated in Figure 4F. Our experimental results confirm that the wall‐climbing robot maintains stable adhesion on surfaces with a maximum roughness Rz of about 50 µm. Furthermore, the flexible acoustic adhesion disk spontaneously adapts to curved surfaces, exhibiting compatibility with convex surfaces (radius ≥ 300 mm) and concave surfaces (radius ≥ 150 mm).

## 3 Discussion

In this study, we present an intelligent climbing robot based on acoustic adhesion that traverses both walls and ceilings while carrying a 100 g payload at up to 20 cm/s. The design addresses two key limitations of acoustic adhesion: vibration–load coupling and sensitivity to substrate tilt. We introduce a ring connection that redistributes disk loading, decouples vibrational excitation from external loads, and increases the stable load capacity by a factor of two. To further reduce tilt sensitivity, we develop an anisotropic origami‐spring connector that helps maintain a sealed, firmly attached disk–substrate interface. Together, these advances provide strong, stable adhesion, enabling the integration of an onboard camera, microcontroller, and battery for wireless control, visual recognition, and intelligent inspection. To quantify and compare the climbing performance of different microrobots using various adhesion mechanisms, we defined a specific power γ, with a unit of power per mass, considering both driving speed and load capacity in Figure 5:

(3) $$\gamma =\alpha \frac{m_{total}}{m_{self}}v=\frac{P_{req}}{m_{self}}\left[specific\;power,\;power\;mass\right]$$

where *m_total_* means the total mass including both the payload and the robot itself *m_self_*. The *v* and *a* denote averaged driving speed and resistive acceleration, respectively. Taking the robot as the reference frame, the power required *P*_req_ at this point can be interpreted as the power necessary to overcome resistance. Wall‐climbing robots are typically designed to ascend vertical surfaces, and their primary performance metric is the work performed against gravity; therefore, under wall‐climbing conditions, it is reasonable to assume that the equivalent acceleration satisfies *a* = *g*, where *g* denotes the gravitational acceleration.

**FIGURE 5:**
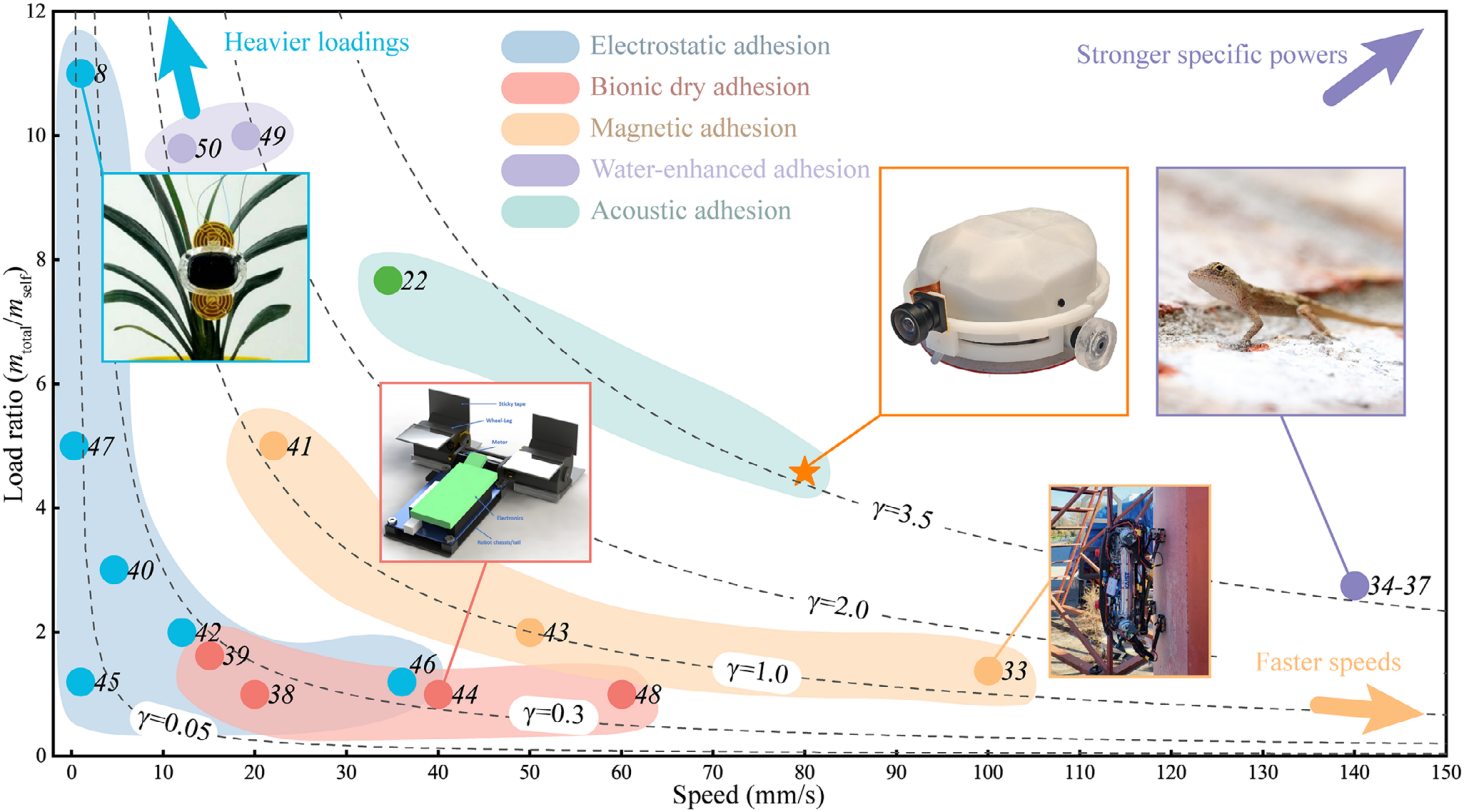
A map compares the performance of wall‐climbing microrobots in terms of both moving speed and load ratio. Specific power γ (dashed line) is defined to quantify and compare the demonstrated powers of various climbing robots based on different adhesion mechanisms [8, 22, 33, 34, 35, 36, 37, 38, 39, 40, 41, 42, 43, 44, 45, 46, 47, 48, 49, 50].

The specific power, γ, principally denotes the power required *P*_req_ for a robot to transport and hold payloads on vertical surfaces against gravity. Compared with conventional evaluations that rely solely on load ratio or speed, the specific power proposed in this paper avoids the pitfall of overemphasizing a single performance metric, which can render the robotic system impractical. For example, focusing exclusively on increasing a robot's payload capacity typically necessitates stronger adhesion to the substrate—reducing detachment efficiency and complicating the design of the locomotion mechanism—thereby reducing its movement speed. Consequently, the product of load‐carrying capacity and speed changes only negligibly, and the overall performance—specific power—remains largely unchanged. High specific power γ therefore reflects innovation in the robot's structure and adhesion mechanisms and serves as an effective comprehensive performance metric for wall‐climbing robots [8, 22, 33, 34, 35, 36, 37, 38, 39, 40, 41, 42, 43, 44, 45, 46, 47, 48, 49, 50] based on different adhesion principles.

Among established adhesion mechanisms for wall‐climbing robots, electrostatic and dry adhesion exhibit relatively low specific power values γ for wall‐climbing robots, at approximately 0.3 W/kg, whereas magnetic adhesion methods increase this value to approximately 1 W/kg. Notably, in addition to acoustic adhesion, water‐enhanced adhesion [49, 50] which utilizes the lubrication and capillary effects of water to achieve adhesion—also achieves specific power γ values approaching or exceeding 2 W/kg. While this method provides strong adhesive force, its application is somewhat limited in many industrial environments (such as high‐temperature turbine blades or electrical equipment) due to the risks of short circuits, corrosion, or contamination.

Compared to similar wall‐climbing robots (indicated by green dots in the figure), our proposed design exhibits superior maneuverability by addressing resonant coupling issues and employing high‐performance origami springs to stabilize the adhesion force during tilting. Overall, the anisotropic origami springs and ring connection enhance the performance of microrobots. Our inspection microrobots achieve a specific power of approximately 3.5 W/kg, exceeding that of many reported climbing robots. The real‐time image recognition network makes the intelligent, small robot particularly well‐suited for in situ equipment inspections.

Smooth surfaces are preferred for forming a stable thin air layer, which is crucial for strong acoustic adhesion. The alloy blades (such as those used in aero‐engines, steam turbines, and gas turbines) typically have clean surfaces and meet the requirements for adhesion generation. Rough, dusty, and porous surfaces are not suitable for the current flexible disk design, primarily because forming a stable air layer is difficult. The variable‐stiffness and multiscale designs facilitate the sealing of a negative‐pressure cavity on non‐smooth surfaces. For example, an adhesion disk with varying stiffness—high at the center and low at the edges—or a sealing lip added around the disk's periphery can compensate for surface roughness and sustain a stable vacuum air layer [51], as shown in Figure S4. Preliminary experimental results indicate that disks with gradually decreasing stiffness and adhesion disks equipped with sealing lips can overcome the limitations imposed by surface roughness and adhere to sandpaper with an Rz of 80 µm. However, the reduced stiffness at the edges leads to greater deformation, thereby compromising the adhesion force. Further experiments and optimization will be undertaken in subsequent research.

Additionally, an excessively small radius of curvature of the substrate can prevent the adhesion disk's edges from lying flat against it, resulting in air leakage. A solution is to use heat pressing to reshape the adhesion disk so that its initial shape more closely conforms to the target substrate. Our validation experiments have demonstrated that pre‐curved adhesion disks fabricated by the thermal creep forming method can adhere to surfaces with smaller radii of curvature (radius = 100 mm), as shown in Figure S5, albeit with reduced adaptability to flat surfaces; this trade‐off will be investigated in future work. This problem can also be addressed by integrating soft robotics techniques, such as a disk deformation mechanism based on the Curved Soft Adaptive Gripper (CSAG) [52, 53, 54], which enables the conforming of high‐curvature surfaces. These potential solutions would expand the applications of such designs to residential settings and collapsed disaster areas.

Currently, the vibrating adhesion disk structure utilizes a polyethylene terephthalate (PET) disk. In high‐temperature scenarios, such as internal inspections of operating high‐temperature turbines, the disk material can be substituted with heat‐resistant organic or inorganic alternatives. Since the underlying adhesion principle is not contingent upon the material's intrinsic properties, it is sufficient to preserve its structural rigidity.

## 4 Conclusion

In this paper, we present an intelligent inspection microrobot that combines high climbing speed with high load capacity in a compact form factor (28 g, 50 mm). The microrobot carries a 100‐g payload while climbing vertical walls at 8 cm/s and traversing inverted ceilings at 20 cm/s, demonstrating strong adhesion performance and agile maneuverability relative to reported wall‐climbing robots. To achieve stable, high‐strength adhesion, we developed a stress‐distributing ring connection and an anisotropic origami‐spring connector. These innovative structures address the key challenges of acoustic adhesion—namely, vibration‐induced failure and application limitations due to stringent assembly and substrate‐angle requirements. Consequently, our robot fully exploits the advantages and inherent characteristics of acoustic adhesion. Theoretical analysis and experimental characterization verified improved acoustic‐adhesion stability and load capacity. With the optimized mechanical design and real‐time vision‐based recognition, the wireless‐controlled robot further demonstrated robust adhesion, autonomy, and surface adaptability in complex environments.

Overall, this study provides an in‐depth exploration of acoustic adhesion and resolves the primary factors limiting its performance. This research advances the practical application of acoustic adhesion, supports the development of vision‐guided feedback control, enhances the performance of wall‐climbing robots, and broadens their scope of application.

## 5 Methods

A control diagram illustrates the control logic, as shown in Figure S6A. The ESP32S3 microcontroller (XIAO ESP32S3 Sense, Seeed Studio, Shenzhen, China) serves as the primary controller for managing the adhesion disk and the robot's posture. It also initially processes video input from the OV2640 camera and wirelessly transmits it to a host computer for visual identification.

The adhesion disk consists of a 50 mm‐diameter, 0.1 mm‐thick PET disk, a ring connection, and an origami spring. The fabrication process is illustrated in Figure S6B. The origami spring is made by folding thick printed paper, while the frames and structures are 3D printed using photosensitive polymers.

We used a 3D line laser scanner (LNX‐8030, Mech Mind, China) and a high‐speed camera (T4040, Phantom, Vision Research Inc., USA) to analyze the changes in the air layer, as shown in Figure S6C.

The stiffness of different springs was measured using a universal tensile machine (WANCE, TSE102A, China, 10 N full scale) with a specified accuracy of ±0.5% of the indicated value. We designed a slide conversion module to determine tangential stiffness (Figure S6D) accurately. Force measurements (Figure S6E) were conducted using the same tension machine. To avoid errors caused by excessive peeling speed, the tensile speed was set to 10 mm/min. The substrate used in the force measurement was an aluminum oxide plate with a surface roughness of 8 µm Ra. The vibration frequency of the acoustic adhesion disk was 200 Hz.

## Author Contributions

J.P., X.X.C., and K.Y. conceived the research plan. X.X.C. and K.Y. constructed the adhesion units and wrote the manuscript. Y.L. designed the robots. Y.M. designed and measured the origami spring. All authors discussed the results throughout the project and edited the manuscript.

## Conflicts of Interest

The authors declare no conflicts of interest.

## Supporting information




**Supporting File 1**: advs78084‐sup‐0001‐S1.mp4.

**Supporting File 2**: advs78084‐sup‐0002‐S2.mp4.

**Supporting File 3**: advs78084‐sup‐0003‐S3.mp4.

**Supporting File 4**: advs78084‐sup‐0004‐S4.mp4.

**Supporting File 5**: advs78084‐sup‐0005‐S5.mp4.

## Data Availability

All data needed to evaluate the conclusions in the paper are present in the paper and/or the Supplementary Materials. Additional data related to this paper may be requested from the authors.
